# Incidence of delirium after non-cardiac surgery in the Chinese elderly population: a systematic review and meta-analysis

**DOI:** 10.3389/fnagi.2023.1188967

**Published:** 2023-06-29

**Authors:** Xiao-Yan Gong, Dong-Jiang Hou, Jing Yang, Jia-li He, Ming-Jin Cai, Wei Wang, Xian-Ying Lu, Jing Gao

**Affiliations:** ^1^School of Nursing, Chengdu University of Traditional Chinese Medicine, Chengdu, Sichuan, China; ^2^School of Medicine and Life Sciences, Chengdu University of Traditional Chinese Medicine, Chengdu, Sichuan, China

**Keywords:** postoperative delirium, elderly, incidence, systematic review, meta-analysis

## Abstract

**Background:**

POD places a heavy burden on the healthcare system as the number of elderly people undergoing surgery is increasing annually because of the aging population. As a large country with a severely aging population, China's elderly population has reached 267 million. There has been no summary analysis of the pooled incidence of POD in the elderly Chinese population.

**Methods:**

Systematic search databases included PubMed, Web of Science, EMBASE, Cochrane Library Databases, China Knowledge Resource Integrated Database (CNKI), Chinese Biomedical Database (CBM), WanFang Database, and Chinese Science and Technology Periodicals (VIP). The retrieval time ranged from the database's establishment to February 8, 2023. The pooled incidence of delirium after non-cardiac surgery was calculated using a random effects model. Meta-regression, subgroup, and sensitivity analyses were used to explore the source of heterogeneity.

**Results:**

A total of 52 studies met the inclusion criteria, involving 18,410 participants. The pooled incidence of delirium after non-cardiac surgery in the elderly Chinese population was 18.6% (95% *CI*: 16.4–20.8%). The meta-regression results revealed anesthesia method and year of publication as a source of heterogeneity. In the subgroup analysis, the gender subgroup revealed a POD incidence of 19.6% (95% *CI*: 16.9–22.3%) in males and 18.3% (95% *CI*: 15.7–20.9%) in females. The year of publication subgroup analysis revealed a POD incidence of 20.3% (95% *CI*: 17.4–23.3%) after 2018 and 14.6 (95% *CI*: 11.6–17.6%) in 2018 and before. In the subgroup of surgical types, the incidence of hip fracture surgery POD was 20.7% (95% *CI*: 17.6–24.3%), the incidence of non-cardiac surgery POD was 18.4% (95% *CI*: 11.8–25.1%), the incidence of orthopedic surgery POD was 16.6% (95% *CI*: 11.8–21.5%), the incidence of abdominal neoplasms surgery POD was 14.3% (95% *CI*: 7.6–21.1%); the incidence of abdominal surgery POD was 13.9% (95% *CI*: 6.4–21.4%). The anesthesia methods subgroup revealed a POD incidence of 21.5% (95% *CI*: 17.9–25.1%) for general anesthesia, 15.0% (95% *CI*: 10.6–19.3%) for intraspinal anesthesia, and 8.3% (95% *CI*: 10.6–19.3%) for regional anesthesia. The measurement tool subgroup revealed a POD incidence of 19.3% (95% *CI*: 16.7–21.9%) with CAM and 16.8% (95% *CI*: 12.6–21.0%) with DSM. The sample size subgroup revealed a POD incidence of 19.4% (95% *CI*: 16.8–22.1%) for patients ≤ 500 and 15.3% (95% *CI*: 11.0–19.7%) for patients > 500. The sensitivity analysis suggested that the pooled incidence of postoperative delirium in this study was stable.

**Conclusion:**

Our systematic review of the incidence of delirium after non-cardiac surgery in elderly Chinese patients revealed a high incidence of postoperative delirium. Except for cardiac surgery, the incidence of postoperative delirium was higher for hip fracture surgery than for other types of surgery. However, this finding must be further explored in future large-sample studies.

**Systematic review registration:**

https://www.crd.york.ac.uk/prospero/, identifier: PROSPERO CRD42023397883.

## Introduction

Postoperative delirium (POD) is a severe surgical complication characterized by inattention, confusion, disorganized perceptual thinking, and emotional variability (Huang et al., 2021). POD usually occurs within 7 days postoperatively, with an exceptionally high incidence on days 1–3 postoperatively, and it occurs because of the vulnerability of brain function to pathophysiological stressors (Jin et al., 2020). The elderly population has a correspondingly increased risk of POD because of the coexistence of multiple diseases and the deterioration of body and cognitive functions (Yürek et al., 2023). As the fastest-growing age group in the world and with increasing numbers of older people undergoing surgery and anesthesia, POD has become a major global health challenge requiring urgent attention (Evered et al., 2021).

Studies have reported that 10–65% of elderly patients undergo delirium after surgery (Deiner et al., 2017; Deeken et al., 2022). The development of delirium triggers a range of poor prognoses and is associated with an increased risk of long-term cognitive impairment and dementia (Ditzel et al., 2023). POD also leads to loss of independence in elderly patients, resulting in delayed functional recovery, more extended hospital stays, and increased mortality after surgery (Oh et al., 2017). In a retrospective study involving 1,260 patients undergoing cardiac surgery, postoperative complications such as myocardial infarction, cerebrovascular accidents, respiratory complications, and infections were significantly more frequent in POD patients (Sugimura et al., 2020). However, in clinical practice, delirium often goes undetected or untreated because of the lack of knowledge of healthcare professionals and the complexity and variability of delirium's clinical presentation, which can have more severe consequences for POD patients (Kong et al., 2022).

The mechanisms underlying the development of POD remain unclear and require further research. Existing theories of POD primarily focus on neuroinflammation, altered neurotransmitters, reduced levels of brain metabolites, oxidative stress, and neuroendocrinology (Li et al., 2022b). Most of these theories are complementary and interact with many fields. In exploring the research process for treating POD, reducing the risk of POD is considered the best way for lowering its incidence (Liu et al., 2022a). Studies have demonstrated that older patients with preoperative dementia, a history of hypnotic drug use, anxiety, and depression are more likely to develop POD than older patients (Guo et al., 2016). Therefore, healthcare professionals must identify people at risk of POD early and develop preventative and intervention strategies.

The DSM-V definition of delirium is now widely accepted and is the gold standard for diagnosing delirium (European Delirium Association and American Delirium Society, 2014). The DSM-V defines delirium as a sudden onset of mood changes with fluctuating levels of consciousness over a short period, with impaired memory, cognitive impairment, disorientation, visual and perceptual disturbances, and language impairment. The most commonly used clinical assessment tools for POD are mainly scales, including the confusion assessment method (CAM), ICU confusion assessment method (CAM-ICU), and nursing delirium screening scale (Nu-DESC) (Zhang and Yin, 2020). Several tests can be used to assess delirium, such as cerebrospinal fluid measurements, electro-encephalogram (EEG) and bispectral index (BIS) monitors (Chew et al., 2022; Schüßler et al., 2023). As delirium research progresses, various objective indicators for assessing and predicting delirium are added, but they are not yet widely used in clinical practice.

Understanding the epidemiological status of POD will draw the attention of healthcare workers to the incidence of POD in older people and facilitate further clinical practice by clinical workers and researchers on POD. China's elderly population has reached 267 million, equivalent to one-fourth of the world's elderly population (Yu and Wang, 2023). With people aged 60 and over accounting for nearly half of the surgical procedures performed annually, POD places a heavy burden on the healthcare system (Fowler et al., 2019). There are many studies on POD in the elderly Chinese population, but they are mostly limited to single surgical procedures. There has been no summary analysis of the combined incidence of POD in the elderly Chinese population. Cardiac surgery is characterized by complex operations and high trauma. Cardiac surgery was excluded from the list to avoid excessive heterogeneity between different surgeries. Therefore, we systematically reviewed the current incidence of POD in non-cardiac surgery in the elderly Chinese population to provide a reference for reporting the epidemiological trends of postoperative delirium and formulating strategic public health decisions.

## Methods

This systematic review and meta-analysis followed the updated 2020 Preferred Reporting Items for Systematic Review and Meta-Analysis (PRISMA) (Page et al., 2021). The protocol was registered in the international prospective register of systematic reviews (CRD42023397883).

### Search strategy

We systematically searched the literature in PubMed, Web of Science, EMBASE, Cochrane Library Databases, China Knowledge Resource Integrated Database (CNKI), Chinese Biomedical Database (CBM), WanFang Database, and Chinese Science and Technology Periodicals (VIP). The retrieval time was from the establishment of the database to January 30, 2023. The search used combination keywords “elder” OR “old” OR “aged” AND “postoperative period” OR “general surgery” OR “Operative” AND “delirium” OR “agitation” OR “postanesthetic excitement” AND “China” OR “Taiwan”. The search strategies used in each database are shown in Appendix A. To determine the comprehensiveness of the search, we manually retrieved references to relevant observational studies and systematic reviews of other potentially eligible studies. We also contacted the corresponding authors to obtain the missing data.

### Study selection

The inclusion criteria were: (a) studies with a cohort or case-control design; (b) participants were aged 60 or above; (c) the assessment tool used The Diagnostic and Statistical Manual of Mental Disorders or confusion assessment method; (d) if study data were duplicated, the study with the largest sample size was selected. The following studies were excluded: (a) reviews, comments, or conference abstracts; (b) those that did not investigate the aims of this review; (c) those with no relevant data; (d) those that did not report the diagnostic criteria for postoperative delirium. After removing duplicate literature, two researchers (GXY and HDJ) screened the article's title, abstract, and full text based on the inclusion and exclusion criteria. If the event of conflicting opinions, a third investigator (GJ) was consulted for settlement.

### Data extraction

Data from the included studies were extracted using prespecified data extraction forms. Data were extracted by one researcher (GXY) and validated by another researcher (YJ). Any disagreement was resolved through negotiated consensus. The extracted data included the author, year of publication, study design, sample size, mean age, gender, type of surgery, measurement tool, the incidence of postoperative delirium, and observational time.

### Quality evaluation

Our systematic review assessed the quality of cohort and case-control studies using the Newcastle-Ottawa scale (NOS) (Wells et al., 2014), and its design, content, and ease of use allowed for the inclusion of quality assessments in the interpretation of meta-analysis results. NOS assigns up to 9 points for minimum bias risk in three domains: (1) group selection (4 points); (2) group comparability (2 points); (3) exposure and outcomes assessment for case-control and cohort studies (3 points). A scale of 0–3 indicates low quality (LQ); 4–6 indicates medium quality (MQ); 7–9 indicates high quality (HQ). Two researchers (GXY and HJL) independently evaluated the quality, then met to discuss the results and reach a consensus on each item on the list.

### Data analysis

The major outcome of this systematic review was the pooled incidence of postoperative delirium in elderly Chinese populations, with 95% confidence intervals (95% *CI*). All data analyses were conducted using Stata version 15.1. Cochran's *Q* and *I*^2^ statistics were used to evaluate the heterogeneity between studies. Heterogeneity was considered to be present when *p* < 0.05. The magnitude of heterogeneity was assessed using the *I*^2^ statistic. Heterogeneity was divided into the following categories: 25–50% (low), 50–75% (moderate), and ≥75% (high) (Higgins et al., 2003). If there was significant heterogeneity, the random-effects model was used to combine the data. Otherwise, the fixed-effects model was used. The results are presented in the form of forest maps. Funnel plot, and Egger's test were used to evaluate the publication bias of research results.

To explore potential sources of heterogeneity, we conducted meta-regression, subgroup analysis, and sensitivity analysis on the included studies. The year of publication, study design, type of surgery, anesthesia method, measurement tool, and sample size were used as covariates to conduct meta-regression to find the source of heterogeneity in the study results. The incidence of delirium and 95% confidence intervals were calculated for subgroups according to gender (male/female), year of publication (After 2018/2018 and before), type of study (abdominal neoplasms surgery/orthopedics surgery/hip fracture surgery, non-cardiac surgery/abdominal surgery), anesthesia methods (general anesthesia/spinal anesthesia/local anesthesia), measurement tool (DSM/CAM), and sample size (< 500/≥500).

## Results

### Study selection

**Figure 1:**
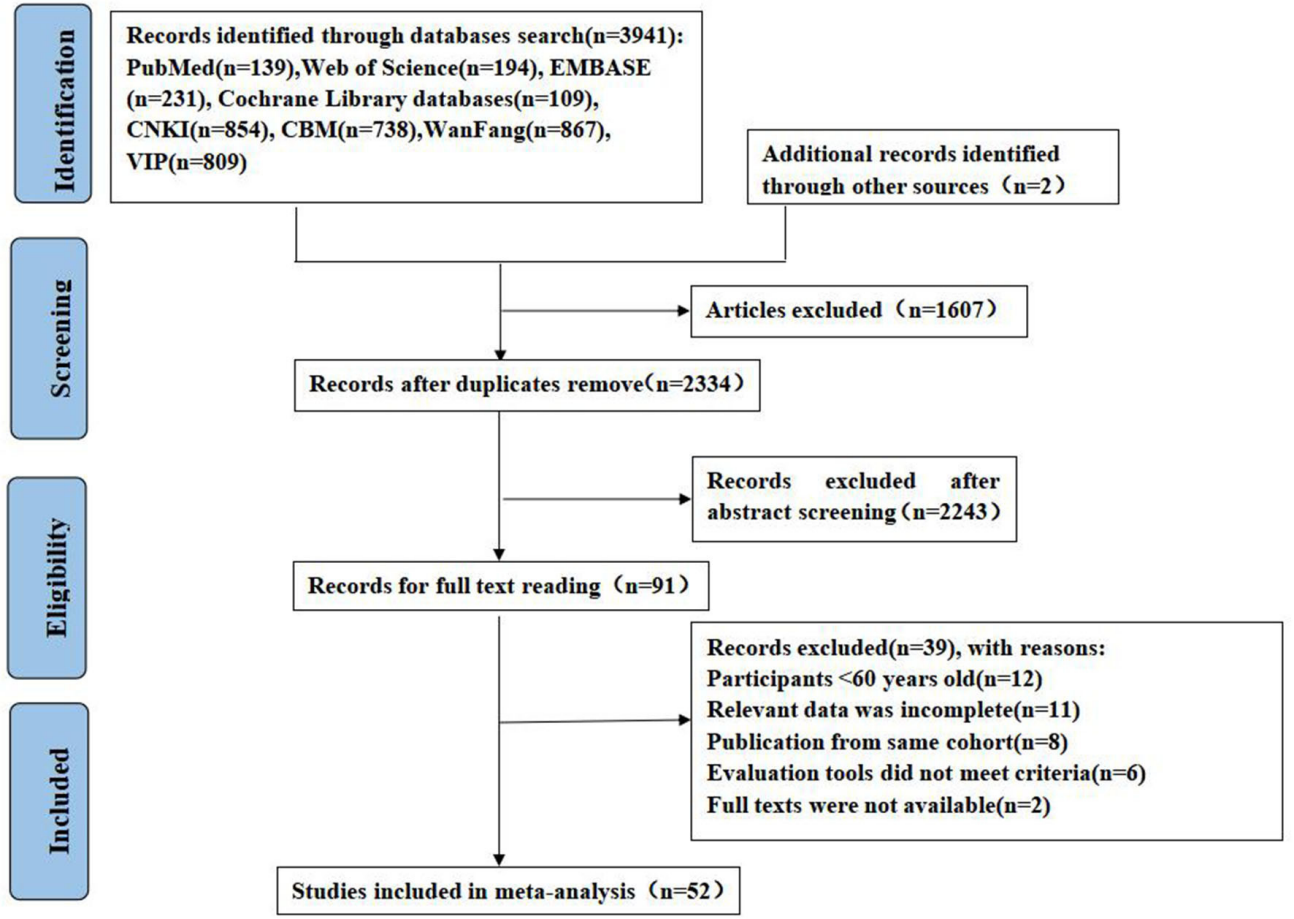
PRISMA flowchart of literature retrieval and selection.

The initial literature search found 3,941 records, of which 1,607 were duplicate. After eliminating duplicate literature, the remaining studies were screened according to the title and abstract, and finally, 91 studies were included for full-text evaluation. After reading the full text, 12 studies were excluded because of age discrepancies in the included population, 11 were excluded because of incomplete reported data, 8 were excluded because of data from the same cohort study, 6 were excluded as the delirium assessment tool did not meet the criteria, and 2 were excluded because of the lack of full-text availability. Finally, 50 studies were included. In addition, two eligible studies were identified from the list of references included in the study. Thus, 52 studies were included in the meta-analysis. The PRISMA flow diagram is shown in Figure 1.

### Study characteristics

A total of 52 studies were included in our systematic review, including 35 prospective cohort studies, 15 retrospective cohort studies, 1 case-control study, and 1 secondary analysis study. We found that 37 of these studies were published after 2018, and 15 were published in 2018 or before. This systematic review included 18,410 elderly patients who had surgery, and 3,200 of them developed delirium afterward. In the included studies, the incidence of postoperative delirium in elderly patients fluctuated from 4.21 to 34.97%. Hip fracture surgery was the most common type of surgery included in the meta-analysis, followed by orthopedic surgery, non-cardiac surgery, abdominal tumor surgery, and abdominal surgery. All included studies have reported clear diagnostic criteria for delirium: 37 studies used the Diagnostic and Statistical Manual of Mental Disorders (DSM) series scale, and 15 studies used the Confusion Assessment Method (CAM) series scale. Most studies (*n* = 31) have reported that POD observation time was generally 3–7 days after surgery, and a few studies (*n* = 19) have not described it in the original article. Table 1 summarizes the characteristics of the 52 included studies.

**Table 1:** Characteristics of included studies.

**References**	**Study design**	**Sample size**	**Mean age (SD)**	**Men (%)**	**Type of surgery**	**Measurement tool**	**Incidence of POD (%)**	**Observational time (day)**	**Quality**
Chen et al. (2022)	RC	270	73.4	65.93%	Abdominal neoplasms	DSM-V	27.41%	7	7
Chu et al. (2016)	PC	544	74.24 (7.92)	43.57%	Orthopedics	DSM-IV-TR	9.56%	NA	7
Cui et al. (2019)	Secondary analysis	620	NA	26.45%	Orthopedics	CAM, CAM-ICU	8.55%	5	6
Feng and Sun (2022)	RC	285	77.68 (6.57)	35.79%	Hip fracture	DSM	4.21%	NA	6
Feng and Yan (2022)	RC	92	74.53 (4.77)	38.04%	Hip fracture	DSM-IV	20.65%	NA	6
Guan et al. (2022)	PC	400	NA	61.50%	Non-cardiac	CAM-ICU	26.75%	3	8
Guan et al. (2020)	PC	246	NA	63.82%	Malignant tumor	DSM-IV	31.30%	3	7
Guo et al. (2016)	PC	572	NA	36.01%	Hip fracture	DSM-IV	20.98%	3	8
Hu et al. (2022)	PC	120	72.4 (4.97)	51.67%	Hip fracture	DSM	12.50%	NA	7
Hu et al. (2019)	PC	217	79.4 (7.9)	35.48%	Hip fracture	CAM	29.03%	NA	7
Ji et al. (2022)	PC	297	69.1 (9.6)	NA	Metrectomy	CAM	25.59%	7	8
Kong et al. (2022)	RC	245	NA	39.18%	Hip fracture	DSM-V	13.06%	NA	6
Kong et al. (2021)	RC	350	69 (5)	74.57%	Oral neoplasms	CAM	30.00%	NA	7
Lai et al. (2023)	PC	345	73	59.71%	Abdominal neoplasms	CAM	5.51%	NA	9
Li et al. (2022a)	RC	384	75.78 (11.62)	30.47%	Hip fracture	CAM	33.33%	3	8
Li et al. (2022b)	PC	196	72.8 (12.5)	58.67%	Hip fracture	CAM	33.67%	3	8
Li et al. (2019)	PC	507	NA	34.32%	Hip fracture	CAM	22.09%	NA	7
Li et al. (2022c)	PC	184	76.15 (7.94)	39.13%	Orthopedics	CAM	32.61%	3	8
Liang et al. (2015)	PC	461	73	42.30%	Orthopedics	CAM	8.03%	NA	6
Liang et al. (2022)	PC	80	NA	51.25%	Non-cardiac	CAM	18.75%	7	7
Liang (2020)	RC	246	75.23 (5.13)	65.45%	Hip fracture	CAM	20.73%	3	7
Liao et al. (2019)	PC	299	NA	52.17%	Non-cardiac surgery	CAM	16.72%	7	7
Lin et al. (2022)	RC	132	76.89 (6.39)	41.67%	Hip fracture	DSM	28.79%	NA	6
Lin et al. (2020)	PC	447	NA	46.98%	Orthopedics	CAM	11.41%	7	7
Liu and Yue (2009)	PC	1,297	NA	49.19%	Non-cardiac	CAM-ICU	7.25%	3	6
Liu (2018)	RC	229	NA	54.59%	Orthopedics	DSM-IV-TR	20.52%	7	7
Liu et al. (2018)	PC	158	75.27 (5.98)	43.67%	Orthopedics	CAM	27.85%	7	7
Liu et al. (2022b)	RC	346	78.17 (7.65)	49.71%	Hip fracture	DSM-V	13.87%	7	7
Liu et al. (2022a)	case–control	889	NA	27.33%	Hip fracture	CAM, CAM-ICU	19.35%	5	6
Liu et al. (2017)	PC	165	78.3 (9.7)	70.30%	Abdominal neoplasms	DSM-IV	13.94%	5	6
Shi et al. (2022)	RC	188	NA	54.26%	Abdominal neoplasms	CAM	13.83%	7	8
Song et al. (2022)	PC	101	73	68.32%	Abdominal operation	DSM-V	15.84%	NA	7
Sun et al. (2022a)	RC	1,051	NA	31.11%	Hip fracture	CAM	14.84%	NA	6
Sun et al. (2022b)	PC	296	NA	53.38%	Orthopedics surgery	CAM-CR	10.47%	NA	7
Tan et al. (2011)	PC	718	NA	NA	Non-cardiac	CAM	11.14%	3	7
Tsai et al. (2022)	PC	345	73	NA	Abdominal operation	CAM	5.51%	7	7
Wang and Wang (2011)	PC	124	NA	65.32%	Non-cardiac	CAM-ICU	33.87%	5	8
Wu and Zhou (2022)	PC	1,215	71.5 (9.9)	54.65%	Orthopedics	CAM	25.02%	3	7
Xiao et al. (2022)	PC	122	NA	31.15%	Orthopedics	CAM	16.39%	3	7
Xing et al. (2020)	PC	163	NA	42.94%	Hip fracture	CAM	34.97%	7	7
Xu et al. (2022b)	PC	267	NA	44.94%	Abdominal operation	CAM	16.48%	2	6
Xu et al. (2022a)	RC	435	67.42 (5.08)	52.18%	Abdominal operation	CAM	18.39%	3	7
Xue et al. (2016)	PC	358	NA	NA	Prostatectomy	DSM-IV	7.82%	NA	8
Yu et al. (2016)	PC	435	NA	32.41%	Hip fracture	CAM	23.45%	3	7
Yuan et al. (2018)	PC	203	80	28.08%	Hip fracture	CAM	9.36%	2	6
Yue et al. (2022)	PC	141	NA	34.75%	Hip fracture	CAM	28.37%	NA	6
Zhang et al. (2017)	PC	382	NA	60.21%	Abdominal neoplasms	CAM	12.04%	7	6
Zhang and Li (2022)	RC	200	NA	67.50%	Hip fracture	CAM	22.50%	NA	7
Zhang and Zhang (2018)	RC	268	77.2 (8.6)	60.07%	Hip fracture	CAM	11.94%	7	7
Zhang et al. (2023)	PC	126	NA	NA	Lower limb surgery	CAM	19.05%	NA	8
Zhou et al. (2020)	PC	187	78.64 (6.92)	56.15%	Tumor operation	CAM	24.60%	NA	7
Zhu et al. (2013)	PC	462	71.5 (4.5)	56.49%	Abdominal neoplasms	CAM	17.97%	3	8

PC, Prospective cohort; RC, Retrospective cohort; NA, Not available; DSM, The Diagnostic and Statistical Manual of Mental Disorders; CAM, The Confusion Assessment Method.

### Quality assessment of included studies

The 52 included studies were evaluated for bias risk and received an average score of 7, indicating that the quality of the included studies was medium to high. The majority of the studies (*n* = 39, 54.2%) were classified as high quality, whereas the remaining 13 studies (45.8%) were classified as medium quality. The most common sources of bias were insufficient cohort follow-up. Furthermore, 17 studies did not report the duration of postoperative delirium observation, and 2 studies had less than the recommended duration of observation. The details of the evaluation process are presented in Appendix B.

### The incidence of postoperative delirium

**Figure 2:**
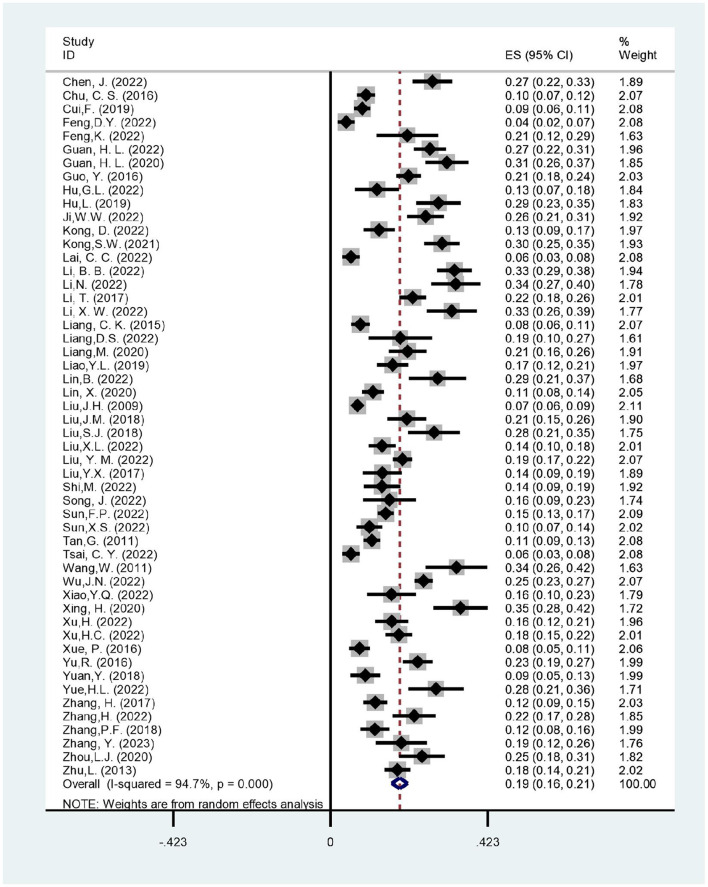
Forest plot of incidence of postoperative delirium.

Our meta-analysis of the incidence of postoperative delirium included 52 studies involving 18,410 participants. In all studies, the incidence of postoperative delirium varied from 4.21 to 34.97%. The included study observed considerable heterogeneity (*I*^2^ = 94.7%, *p* < 0.001). Therefore, using the random effect model, the results demonstrated that the pooled incidence of delirium after non-cardiac surgery in the elderly Chinese population was 18.6% (95% *CI*: 16.4–20.8%) (Figure 2).

### Meta-regression analysis

Meta-regression is an analytical method used to explore the size and source of heterogeneity among different studies, which is a fusion of meta-analysis and linear regression principles (Sutton and Higgins, 2008). To investigate the heterogeneous sources of POD incidence in the elderly Chinese population, we used meta-regression with the year of publication, study design, type of surgery, anesthesia method, measurement tool, and sample size as covariates (Table 2). The meta-regression results revealed that study design (*p* = 0.577), type of surgery (*p* = 0.950), measurement tools (*p* = 0.367), and sample size (*p* = 0.186) had no significant correlation with POD incidence in the elderly Chinese population. The year of publication (*p* = 0.023) and anesthesia method (*p* = 0.007) may affect POD incidence among the elderly Chinese population.

**Table 2:** Meta-regression analysis of included studies.

**Covariates**	**β**	***SE***	**95% *CI***	***P***
Year of publication	0.008	0.004	0.001 to 0.015	0.023
Study design	0.011	0.021	−0.052 to 0.029	0.577
Type of surgery	< 0.001	0.006	−0.011 to 0.011	0.950
Anesthesia method	−0.065	0.024	−0.113 to −0.018	0.007
Measurement tool	0.024	0.027	−0.028 to 0.077	0.367
Sample size	−0.004	0.030	−0.101 to 0.186	0.186

### Subgroup analysis and sensitivity analysis

Subgroup analysis was used to investigate study heterogeneity. Subgroup analyses were performed according to gender, year of publication, type of surgery, method of anesthesia, measurement tool, and sample size (Table 3). The gender subgroup analysis included 46 studies, and the results revealed that the pooled incidence of postoperative delirium was 19.6% in males (95% *CI*: 16.9–22.3%) and 18.3% in females (95% *CI*: 15.7–20.9%). The subgroup analysis by year of publication revealed a pooled incidence of postoperative delirium of 20.3% (95% *CI*: 17.4–23.3%) after 2018, higher than the pooled incidence of 14.6% (95% *CI*: 11.6–17.6%) in 2018 and before. In the subgroup of surgical types, the pooled incidence of postoperative delirium after hip surgery was the highest [20% (95% *CI*: 17.6–24.3%)], and abdominal surgery had the lowest incidence [13.9% (95% *CI*: 6.4–21.4%)]. The subgroup analysis of anesthesia methods demonstrated that the POD incidence was the highest in patients with general anesthesia [21.5% (95% *CI*: 17.9–25.1%)], higher than that in patients with spinal anesthesia [15.0% (95% *CI*: 10.6–19.3%)], and the lowest in patients with local anesthesia [8.3% (95% *CI*: 1.3–15.3%)]. In the measurement tool subgroup, the pooled incidence of postoperative delirium was 19.3% (95% *CI*: 16.7–21.9%) with CAM and 16.8% (95% *CI*: 12.6–21.0%) with DSM. The subgroup analysis of sample size revealed that the pooled incidence of postoperative delirium was 19.4% (95% *CI*: 16.8–22.1%) for patients ≤ 500 and 15.3% (95% *CI*: 11.0–19.7%) for patients > 500.

**Table 3:** Subgroup analysis results of included studies.

**Subgroups**	**Number of studies**	**Sample size**	**Heterogeneity**		**Model**	**incidence**	**95% *CI***
			*I*^2^ **(%)**	***p***			
**Gender**							
Male	46	7,454	90.5	< 0.001	Random	19.6%	16.9–22.3%
Female	46	8,677	92.3	< 0.001	Random	18.3%	15.7–20.9%
**Year of publication**							
After 2018	37	11,651	94.8	< 0.001	Random	20.3%	17.4–23.3%
2018 and before	15	6,579	92.9	< 0.001	Random	14.6%	11.6–17.6%
**Type of surgery**							
Abdominal neoplasms surgery	5	1,350	93.3	< 0.001	Random	14.3%	7.6–21.1%
Orthopedics surgery	10	4,276	95.3	< 0.001	Random	16.6%	11.8–21.5%
Hip fracture surgery	21	6,879	93.6	< 0.001	Random	20.7%	17.1–24.3%
Non-cardiac surgery	6	2,918	95.7	< 0.001	Random	18.4%	11.8–25.1%
Abdominal surgery	4	1,148	93.1	< 0.001	Random	13.9%	6.4–21.4%
**Anesthesia method**							
General anesthesia	24	4,391	90.0	< 0.001	Random	21.5%	17.9–25.1%
Intraspinal anesthesia	16	3,054	93.2	< 0.001	Random	15.0%	10.6–19.3%
Regional anesthesia	4	517	91.5	< 0.001	Random	8.3%	1.3–15.3%
**Measurement tool**							
DSM	14	3,705	93.2	< 0.001	Random	16.8%	12.6–21.0%
CAM	38	14,705	95.1	< 0.001	Random	19.3%	16.7–21.9%
**Sample size**							
≤ 500	43	10,997	93.9	< 0.001	Random	19.4%	16.8–22.1%
> 500	9	7,413	96.9	< 0.001	Random	15.3%	11.0–19.7%

Sensitivity analysis was performed by eliminating individual studies one at a time. The incidence of postoperative delirium fluctuated between 18.4 and 18.9%, with little difference between the obtained results and the overall incidence, implying that our findings were stable. The details of the sensitivity analysis are presented in Appendix C.

### Publication bias

**Figure 3:**
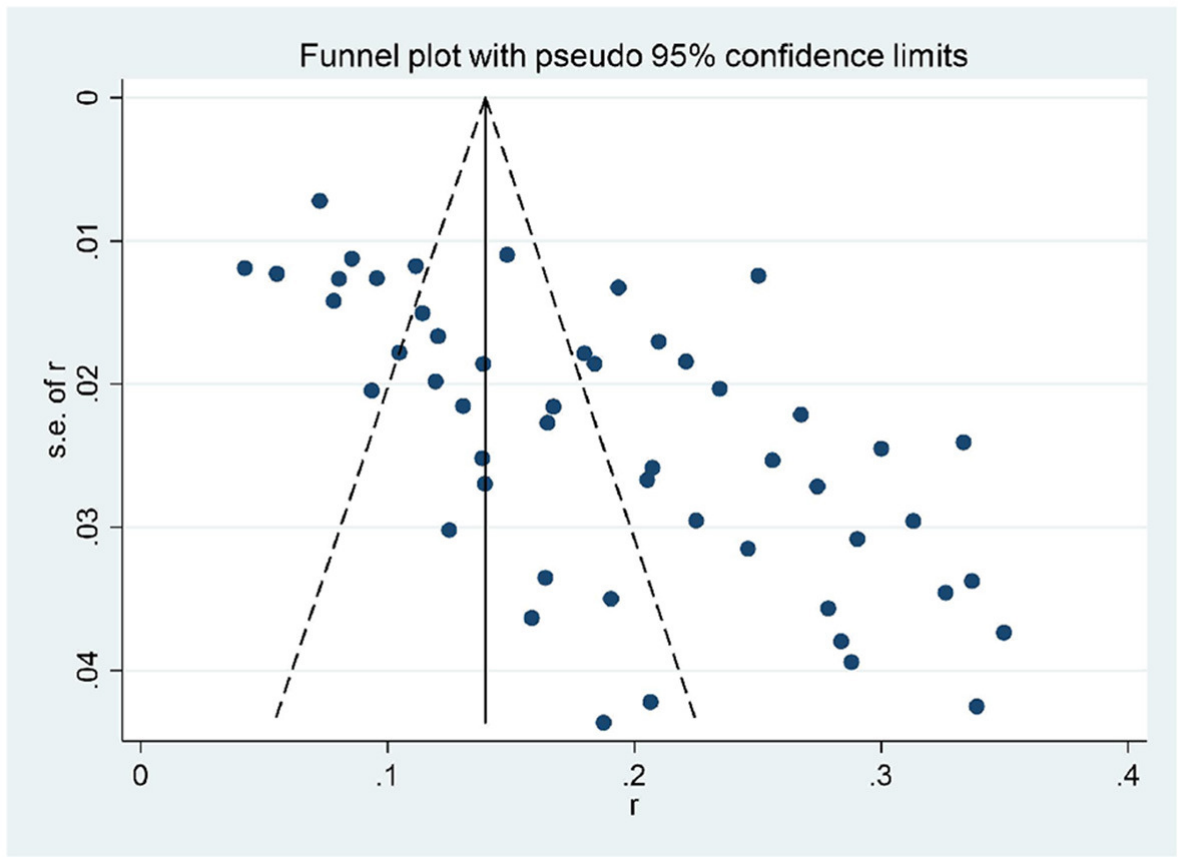
Funnel plot with pseudo 95% confidence limits.

We used the funnel chart and Egger's test to test the publication bias of the pooled incidence of postoperative delirium. The funnel plot revealed significant asymmetry upon visual inspection (Figure 3). Egger's test (*P* = 0.157) revealed no evidence of publication bias.

## Discussion

To our knowledge, this study is the first meta-analysis to include the incidence of delirium after non-cardiac surgery in the elderly Chinese population. Elderly patients undergoing non-cardiac surgery were included in this study. A total of 52 studies—with 18,410 participants aged 60 and over—were included. The risk of bias in 52 included studies was assessed, and the average score for 52 studies was 7. The majority of the studies (*n* = 39, 54.2%) were classified as high quality, whereas the remaining 13 studies (45.8%) were classified as medium quality. According to the quality evaluation results, the most common causes of bias were insufficient cohort follow-up. Our meta-analysis found that the pooled incidence of delirium after non-cardiac surgery was 18.6% (95% *CI*: 16.4–20.8%, *I*^2^ = 94.7%, *p* < 0.001). Because of the high level of heterogeneity in this study, meta-regression, subgroup analysis, and sensitivity analysis were used to investigate the sources of heterogeneity. The publication anesthesia method and year of the study was discovered to be the source of heterogeneity through meta-regression, but the subgroup and sensitivity analyses failed to identify the source of heterogeneity.

According to our systematic review, the incidence of non-cardiac postoperative delirium in the elderly Chinese population was relatively high (18.6%), which is higher than the incidence of postoperative delirium in elderly patients in a multicenter study in the UK (16.3%) (Geriatric Medicine Research Collaborative, 2023) but lower than the incidence of delirium in elderly population after cardiac surgery (23%) (Tilley et al., 2018). The incidence of POD in the elderly population in different regions and countries is affected by medical conditions, environment, and other factors, and the results differ (Brooks Carthon et al., 2013). Studies have demonstrated that POD incidence is highly correlated with the type of surgery. The incidence of POD varies with different surgical characteristics, and the incidence of POD is usually low in minor surgery and day surgery (Iamaroon et al., 2020). American scholar Berian et al. (2018) investigated the occurrence of POD in 20,212 elderly surgical patients over 65 years old from 30 hospitals and found that the incidence of POD in elderly surgical patients over 65 years old was 12.01%; the operations with POD incidence from high to low were as follows: thoracic surgery 13.65%, general surgery 12.97%, orthopedics surgery 12.96%, vascular surgery 11.38%, neurosurgery 7.56%, otorhinolaryngology 7.09%, urology 6.57%, and gynecology 4.71%. Notably, the incidence of postoperative delirium was higher in thoracic surgery, general surgery, and orthopedic surgery compared to other surgery types. This finding is similar to ours, although our systematic review did not include thoracic surgery.

The difference in the incidence of postoperative delirium among subgroups was obtained through subgroup analysis. In the subgroup analysis of gender, the incidence of postoperative delirium of elderly males was higher than that of elderly female patients, consistent with the research conclusion of Gao et al. (2022). This finding may be related to the more frequent alcohol consumption by men among the elderly Chinese population (Liu et al., 2019). Furthermore, prospective studies on the relationship between alcohol abuse and POD have confirmed that alcohol abuse is an independent risk factor for POD (Sousa et al., 2017). Laboratory tests on POD found that POD patients exhibited an average erythrocyte volume > 95.0 fl, a marker often associated with alcoholism (Findley et al., 2010). In a subgroup analysis of the year of publication, the incidence of postoperative delirium in the elderly population was higher after 2018 than in 2018 and before. According to the publication trend of delirium research in China, delirium research has entered a period of rapid development after 2018. During 2019–2020, the number of articles published on studies related to postoperative delirium in China increased rapidly, with a total of 687 articles, representing ~31% of the total number of articles published on the topic of delirium (You et al., 2022). Concurrently, Chinese healthcare professionals are becoming aware of and concerned about delirium, which may improve its detection rate. In the subgroup analysis of the type of surgery, the highest incidence of postoperative delirium was seen in older people with hip fractures, followed by non-cardiac surgery, orthopedic surgery, abdominal oncology surgery, and abdominal surgery. Older people often have osteoporosis and are prone to hip fractures after a fall (Hata et al., 2023). Hip fractures are highly disabling and fatal (Koso et al., 2018). As surgical and anesthetic techniques continue to develop, more patients opt for surgical treatment. However, while morbidity and mortality rates are decreasing, the incidence of postoperative delirium in elderly people is increasing (Albanese et al., 2022). The subgroup analysis of anesthesia methods revealed that the incidence of general anesthesia (21.5%) was higher than that of intravertebral anesthesia (15.0), and the incidence of local anesthesia (8.3) was the lowest, which is consistent with the conclusions of Zhuang et al. (2022). This finding could be attributed to anesthetic drugs that can affect the regulation of neurotransmitters and receptors in the central system of patients and can affect the postoperative cognitive dysfunction and mental state of patients (Sieber et al., 2019). The relationship between the mode of anesthesia and delirium is complex, and the mechanism has not been fully elucidated. However, POD is more likely to occur during general anesthesia when multiple anesthetic agents are required to maintain intraoperative sedation than during intraspinal anesthesia. The depth of intraoperative sedation is thought to affect cognitive function, and the shallower the depth of anesthesia, the lower the incidence of POD (Evered and Silbert, 2018). In the subgroup analysis of measurement tools, studies applying the CAM had a higher incidence of postoperative delirium, consistent with the findings of Gao et al. (2022). In a study by Inouye et al., the sensitivity and specificity of CAM for the diagnosis of delirium and specificity were >95%. The advantages of the CAM scale are that the items are simple and clear; the process can be completed in < 5 min; the scale can be used in emergency, postoperative, and mixed settings (De and Wand, 2015). It is the most widely used screening tool for delirium in general hospitals, as it performs well in emergency, post-operative, and mixed hospital settings. It is challenging to differentiate delirium from other psychiatric disorders (Mulkey et al., 2018), which is a limitation. In the subgroup analysis of the sample size, the incidence of postoperative delirium was higher in studies with sample sizes ≤ 500. Studies with larger sample sizes have included more types of surgery, whereas studies with a single surgery type tend to focus more on patient groups with a high incidence of delirium, possibly resulting in a high incidence of postoperative delirium in studies with sample sizes ≤ 500 (Liu and Yue, 2009).

### Clinical and research implications

Current evidence from epidemiological studies on POD suggests that the incidence of POD in elderly patients is highly heterogeneous, ranging from ~5 to 70% (Avidan et al., 2017). The main reason for this high incidence of POD is the temporal trends in elderly patients and the variation in incidence between populations, countries, and even between regions of the same country, and this variation persists even after studies have controlled for confounding factors, such as type of surgery and age (Takeuchi et al., 2012). The research implications of our systematic review are as follows. First, studies on the systematic evaluation of POD incidence have mainly focused on one or a few types of surgery, and few studies have examined the differences in the incidence of POD among different types of surgery in the same region. Second, no systematic review on the incidence of postoperative delirium in elderly Chinese people has been published. The present study included studies on the incidence of POD in elderly Chinese patients undergoing non-cardiac surgery from a comprehensive and holistic perspective, reducing the differences in cultural background, the healthcare environment, and patients' consultation habits between different countries and regions, which has important practical significance and clinical guidance value. Finally, our study not only reports the incidence of non-cardiac surgical POD in elderly Chinese patients, effectively guiding the early identification of high-risk groups, but also provides a more comprehensive pre-study basis for POD etiology studies. Moreover, because of China's large elderly population of 267 million, this systematic review can provide an essential reference for the global epidemiological status of POD and public health policy formulation.

### Strengths and limitations

The main strength of our review is that we conducted a comprehensive literature search without language limitation through screening independently, extracting data carefully, evaluating quality strictly, and supplementing it with the list of research references. The funnel chart and Egger's test were used to evaluate the publication bias of the research results. Meta-regression, subgroup analysis, and sensitivity analysis were comprehensively used to explore the source of heterogeneity. Our study also has limitations. The meta-analysis in our study was highly heterogeneous, with only the meta-regression results demonstrating year of publication and anesthesia method as sources of heterogeneity and failing to identify other sources of heterogeneity. We estimate that the source of heterogeneity may be related to differences in literacy, narcotic drugs and the level of medical care in the area of residence of older people. Detailed demographic data would be more helpful in assessing the incidence characteristics of postoperative delirium in elderly Chinese people. Not all included studies have provided demographic characteristics of participants; therefore, We did not conduct more detailed subgroup analyses for more groups. In future delirium-related studies, there is a need to continue refining baseline data on older adults.

## Conclusion

Our systematic review of the incidence of delirium after non-cardiac surgery in elderly Chinese patients revealed a high incidence of postoperative delirium, which significantly burdens the healthcare system. There are more studies on postoperative delirium in older adults, but the baseline characteristics of older adults are less comprehensively described, and systematic reviews are more heterogenous because of the influence of various factors. Based on the current research, it appears that among the various types of surgery, except for cardiac surgery, hip fracture surgery has a higher incidence of postoperative delirium, which should be confirmed through future large sample studies.

## Data availability statement

The original contributions presented in the study are included in the article/Supplementary material, further inquiries can be directed to the corresponding authors.

## Author contributions

X-YG, D-JH, and JY contributed to the drafting of the manuscript. X-YG, D-JH, and JG contributed to the study design. X-YG, J-lH, WW, and X-YL contributed to statistical analysis, verification of data, and interpretation of data. X-YG, D-JH, JY, and X-YL contributed to the critical revision of the manuscript for important intellectual content. JG and JY contributed to the concept, supervision, and funding. All authors contributed to the article and approved the submitted version.
