# Supplementary material for: Incidence of delirium after non-cardiac surgery in the Chinese elderly population: a systematic review and meta-analysis
Source: Front Aging Neurosci. 2023 Jun 29;15:1188967. doi: 10.3389/fnagi.2023.1188967 (PMC10346854; doi:10.3389/fnagi.2023.1188967)
Supplement: Supplementary file 1 [file Table_1.DOCX]

**Appendix A：The search strategy used in each database**

1. **Pubmed**

#1 ((Aged[MeSH Terms]) OR (Aging[MeSH Terms])) OR (Geriatrics[MeSH Terms])

#2 ((((((((((((((Aging[Title/Abstract]) OR (Senescence[Title/Abstract])) OR (Biological Aging[Title/Abstract])) OR (elderly[Title/Abstract])) OR (Geriatric*[Title/Abstract])) OR (gerontology[Title/Abstract])) OR (elder[Title/Abstract])) OR (Pensioner[Title/Abstract])) OR (Veteran[Title/Abstract])) OR (Older[Title/Abstract])) OR (Senile[Title/Abstract])) OR (Senility[Title/Abstract])) OR (Old[Title/Abstract])) OR (Ancient[Title/Abstract])) OR (Advanced Age[Title/Abstract])

#3 #1 OR #2

#4 Postoperative Period [MeSH Terms] OR General Surgery[MeSH Terms] OR Surgical Procedures, Operative [MeSH Terms]

#5 ((((((((((((((((((((postoperative[Title/Abstract]) OR (Period, Postoperative[Title/Abstract])) OR (Periods, Postoperative[Title/Abstract])) OR (Postoperative Periods[Title/Abstract])) OR (Surgery, General[Title/Abstract])) OR (Surgery[Title/Abstract])) OR (Operative Procedures[Title/Abstract])) OR (Operative Procedure[Title/Abstract])) OR (Procedure, Operative[Title/Abstract])) OR (Procedures, Operative[Title/Abstract])) OR (Surgical Procedure, Operative[Title/Abstract])) OR (Operative Surgical Procedures[Title/Abstract])) OR (Procedure, Operative Surgical[Title/Abstract])) OR (Procedures, Operative Surgical[Title/Abstract])) OR (Surgical Procedures[Title/Abstract])) OR (Procedure, Surgical[Title/Abstract])) OR (Procedures, Surgical[Title/Abstract])) OR (Surgical Procedure[Title/Abstract])) OR (Operative Surgical Procedure[Title/Abstract])) OR (Operative[Title/Abstract])) OR (Operation[Title/Abstract])

#6 #4 OR #5

#7 Delirium [MeSH Terms] OR Emergence Delirium [MeSH Terms] 12,176

#8 ((((((((((((((((((((((((Subacute Delirium[Title/Abstract]) OR (Delirium, Subacute[Title/Abstract])) OR (Delirium[Title/Abstract])) OR (Subacute Deliriums[Title/Abstract])) OR (Delirium of Mixed Origin[Title/Abstract])) OR (Mixed Origin Delirium[Title/Abstract])) OR (Mixed Origin Deliriums[Title/Abstract])) OR (Delirium, Emergence[Title/Abstract])) OR (Emergence Agitation[Title/Abstract])) OR (Agitation, Emergence[Title/Abstract])) OR (Agitations, Emergence[Title/Abstract])) OR (Post-Operative Delirium[Title/Abstract])) OR (Delirium, Post-Operative[Title/Abstract])) OR (Post Operative Delirium[Title/Abstract])) OR (Postanesthetic Excitement[Title/Abstract])) OR (Excitement, Postanesthetic[Title/Abstract])) OR (Anesthesia Emergence Delirium[Title/Abstract])) OR (Delirium, Anesthesia Emergence[Title/Abstract])) OR (Emergence Delirium, Anesthesia[Title/Abstract])) OR (Postoperative Delirium[Title/Abstract])) OR (Delirium, Postoperative[Title/Abstract])) OR (Agitated Emergence[Title/Abstract])) OR (Agitated Emergence[Title/Abstract])) OR (Emergence Excitement[Title/Abstract])) OR (Excitement, Emergence[Title/Abstract])

#9 #7 OR #8

#10 China [MeSH Terms] OR Taiwan [MeSH Terms]

#11((((((((((((China[Text Word]) OR (Chinese[Text Word])) OR (People's Republic of China[Text Word])) OR (Mainland China[Text Word])) OR (Sinkiang[Text Word])) OR (Inner Mongolia[Text Word])) OR (Manchuria[Text Word])) OR (Beijing[Text Word])) OR (Hong Kong[Text Word])) OR (Macau[Text Word])) OR (Tibet[Text Word])) OR (Formosa[Text Word])) OR (Taiwan[Text Word])

#12 #10 OR #11

#13 #3 AND #6 AND #9 AND #12

**A total of 139 studies were retrieved**

1. **Embase**

#1 'Aged'/exp OR 'Aging'/exp OR 'Geriatrics'/exp

#2 'Aging':ab,ti OR 'Senescence':ab,ti OR 'Biological Aging':ab,ti OR 'elderly':ab,ti OR 'Geriatric*':ab,ti OR 'gerontology':ab,ti OR 'elder':ab,ti OR 'Pensioner':ab,ti OR 'Veteran':ab,ti OR 'Older':ab,ti OR 'Senile':ab,ti OR 'Senility':ab,ti OR 'Old':ab,ti OR 'Ancient':ab,ti OR 'Advanced Age':ab,ti

#3 #1 OR #2

#4 'Postoperative Period'/exp OR 'General Surgery'/exp OR 'Surgical Procedures, Operative'/exp

#5 'postoperative':ab,ti OR 'Period, Postoperative':ab,ti OR 'Periods, Postoperative':ab,ti OR 'Postoperative Periods':ab,ti OR 'Surgery, General':ab,ti OR 'Surgery':ab,ti OR 'Operative Procedures':ab,ti OR 'Operative Procedure':ab,ti OR 'Procedure, Operative':ab,ti OR 'Procedures, Operative':ab,ti OR 'Surgical Procedure, Operative':ab,ti OR 'Operative Surgical Procedures':ab,ti OR 'Procedure, Operative Surgical':ab,ti OR 'Procedures, Operative Surgical':ab,ti OR 'Surgical Procedures':ab,ti OR 'Procedure, Surgical':ab,ti OR 'Procedures, Surgical':ab,ti OR 'Surgical Procedure':ab,ti OR 'Operative Surgical Procedure':ab,ti OR 'Operative':ab,ti OR 'Operation':ab,ti

#6 #4 OR #5

#7 'Delirium'/exp OR 'Emergence Delirium'/exp

#8 'Subacute Delirium':ab,ti OR 'Delirium, Subacute':ab,ti OR 'Delirium':ab,ti OR 'Subacute Deliriums':ab,ti OR 'Delirium of Mixed Origin':ab,ti OR 'Mixed Origin Delirium':ab,ti OR 'Mixed Origin Delirium':ab,ti OR 'Delirium, Emergence':ab,ti OR 'Emergence Agitation':ab,ti OR 'Agitation, Emergence':ab,ti OR 'Agitations, Emergence':ab,ti OR 'Post-Operative Delirium':ab,ti OR 'Delirium, Post-Operative':ab,ti OR 'Post Operative Delirium':ab,ti OR 'Post Operative Delirium':ab,ti OR 'Excitement, Postanesthetic':ab,ti OR 'Anesthesia Emergence Delirium':ab,ti OR 'Delirium, Anesthesia Emergence':ab,ti OR 'Emergence Delirium, Anesthesia':ab,ti OR 'Postoperative Delirium':ab,ti OR 'Delirium, Postoperative':ab,ti OR 'Agitated Emergence':ab,ti OR 'Agitated Emergence':ab,ti OR 'Emergence Excitement':ab,ti OR 'Emergence Excitement':ab,ti

#9 #7 OR #8

#10 'China'/exp OR 'Taiwan'/exp

#11 China OR Chinese OR People's Republic of China OR Mainland China OR Sinkiang OR Inner Mongolia OR Manchuria OR Beijing OR Hong Kong OR Macau OR Tibet OR Formosa OR Taiwan

#12 #10 OR #11

#13 #3 AND #6 AND #9 AND #12

**A total of 231 studies were retrieved**

**3.Web of Science**

#1 Aged OR Aging OR Geriatrics OR Senescence OR Biological Aging OR elderly OR Geriatric* OR gerontology OR elder OR Pensioner OR Veteran OR Older OR Senile OR Senility OR Old OR Ancient OR Advanced Age [Title]

#2 Postoperative Period OR General Surgery OR Surgical Procedures, Operative OR postoperative OR Period, Postoperative OR Periods, Postoperative OR Postoperative Periods OR Surgery, General OR Surgery OR Operative Procedures OR Operative Procedure OR Procedure, Operative OR Procedures, Operative OR Surgical Procedure, Operative OR Operative Surgical Procedures OR Procedure, Operative Surgical OR Procedures, Operative Surgical OR Surgical Procedures OR Procedure, Surgical OR Procedures, Surgical OR Surgical Procedure OR Operative Surgical Procedure OR Operative OR Operation[Abstract]

#3 Delirium OR Emergence Delirium OR Subacute Delirium OR Delirium, Subacute OR Delirium OR Subacute Deliriums OR Delirium of Mixed Origin OR Mixed Origin Delirium OR Mixed Origin Deliriums OR Delirium, Emergence OR Emergence Agitation OR Agitation, Emergence OR Agitations, Emergence OR Post-Operative Delirium OR Delirium, Post-Operative OR Post Operative Delirium OR Postanesthetic Excitement OR Excitement, Postanesthetic OR Anesthesia Emergence Delirium OR Delirium, Anesthesia Emergence OR Emergence Delirium, Anesthesia OR Postoperative Delirium OR Delirium, Postoperative OR Agitated Emergence OR Agitated Emergence OR Emergence Excitement OR Excitement, Emergence[Title]

#4 China OR Chinese OR People's Republic of China OR Mainland China OR Sinkiang OR Inner Mongolia OR Manchuria OR Beijing OR Hong Kong OR Macau OR Tibet OR Formosa OR Taiwan

#5 #1 AND #2 AND #3 AND #4

**A total of 194 studies were retrieved**

1. **Cochrane**

#1 Aged OR Aging OR Geriatrics OR Senescence OR Biological Aging OR elderly OR Geriatric* OR gerontology OR elder OR Pensioner OR Veteran OR Older OR Senile OR Senility OR Old OR Ancient OR Advanced Age [Title]

#2 Postoperative Period OR General Surgery OR Surgical Procedures, Operative OR postoperative OR Period, Postoperative OR Periods, Postoperative OR Postoperative Periods OR Surgery, General OR Surgery OR Operative Procedures OR Operative Procedure OR Procedure, Operative OR Procedures, Operative OR Surgical Procedure, Operative OR Operative Surgical Procedures OR Procedure, Operative Surgical OR Procedures, Operative Surgical OR Surgical Procedures OR Procedure, Surgical OR Procedures, Surgical OR Surgical Procedure OR Operative Surgical Procedure OR Operative OR Operation[Abstract]

#3 Delirium OR Emergence Delirium OR Subacute Delirium OR Delirium, Subacute OR Delirium OR Subacute Deliriums OR Delirium of Mixed Origin OR Mixed Origin Delirium OR Mixed Origin Deliriums OR Delirium, Emergence OR Emergence Agitation OR Agitation, Emergence OR Agitations, Emergence OR Post-Operative Delirium OR Delirium, Post-Operative OR Post Operative Delirium OR Postanesthetic Excitement OR Excitement, Postanesthetic OR Anesthesia Emergence Delirium OR Delirium, Anesthesia Emergence OR Emergence Delirium, Anesthesia OR Postoperative Delirium OR Delirium, Postoperative OR Agitated Emergence OR Agitated Emergence OR Emergence Excitement OR Excitement, Emergence[Title]

#4 China OR Chinese OR People's Republic of China OR Mainland China OR Sinkiang OR Inner Mongolia OR Manchuria OR Beijing OR Hong Kong OR Macau OR Tibet OR Formosa OR Taiwan[Text word]

#5 #1 AND #2 AND #3 AND #4

**A total of 109 studies were retrieved**

1. **知网**

TI=('老人'+ '老年'+ '老年人') AND TI=('外科手术'+'手术后期间'+'手术'+'术后') AND TI=('谵妄'+'谵语'+'精神错乱'+'ICU综合征'+'急性精神病状态'+'急性混乱状态'+'加护病房症候群'+'代谢性脑部疾病'+'中毒性脑部疾病') AND AB=('患病率'+'发病率'+'流行病学'+'流行病学研究'+'现患调查'+'发病率研究'+'发生率')

**A total of 854 studies were retrieved**

**6.万方数据库检索策略**

题名:("老年人" or "老人" or "老年") and 题名:("外科手术"or "手术后期间" or "手术" or "术后" ) and 题名:("谵妄"or"谵语"or"精神错乱"or"ICU综合征"or"急性精神病状态"or"急性混乱状态"or"加护病房症候群"or"代谢性脑部疾病"or"中毒性脑部疾病") and 主题:("患病率"or"发病率"or"流行病学"or"流行病学研究"or"现患调查"or"发病率研究"or"发生率")

**A total of 867 studies were retrieved**

**7.维普中文期刊数据库检索策略**

(T=老人 OR T=老年 OR T=老年人) AND (T=外科手术 OR T=手术后期间 OR T=手术 OR T=术后) AND (T=谵妄 OR T=谵语 OR T=精神错乱 OR T=ICR综合征 OR T=急性精神病状态 OR T=急性混乱状态 OR T=加护病房症候群 OR T=代谢性脑部疾病 OR T=中毒性脑部疾病) AND (R=患病率 OR R=发病率 OR R=流行病学 OR R=流行病学研究 OR R=现患调查 OR R=发病率研究 OR R=发生率)

**A total of 809 studies were retrieved**

1. **CBM**

#1 "老年人"[标题:智能] OR "老人"[标题:智能] OR "老年"[标题:智能]

#2 "外科手术"[标题:智能] OR "手术后期间"[标题:智能] OR "手术"[标题:智能] OR "术后"[标题:智能]

#3 "谵妄"[标题:智能] OR "谵语"[标题:智能] OR "精神错乱"[标题:智能] OR "ICR综合征"[标题:智能] OR "急性精神病状态"[标题:智能] OR "急性混乱状态"[标题:智能] OR "加护病房症候群"[标题:智能] OR "代谢性脑部疾病"[标题:智能] OR "中毒性脑部疾病"[标题:智能]

#4 "患病率"[摘要:智能] OR "发病率"[摘要:智能] OR "流行病学"[摘要:智能] OR "流行病学研究"[摘要:智能] OR "现患调查"[摘要:智能] OR "发病率研究"[摘要:智能] OR "发生率"[摘要:智能]

**A total of 738 studies were retrieved**
