# Supplementary material for: Incidence of delirium after non-cardiac surgery in the Chinese elderly population: a systematic review and meta-analysis
Source: Front Aging Neurosci. 2023 Jun 29;15:1188967. doi: 10.3389/fnagi.2023.1188967 (PMC10346854; doi:10.3389/fnagi.2023.1188967)
Supplement: Supplementary file 2 [file Table_2.DOCX]

**Appendix B：Assessment of the risk of bias in the included studies**

Table 1 Quality evaluation of cohort studies

| Author/Year | 1 | 2 | 3 | 4 | 5 | 6 | 7 | 8 | Score |
| --- | --- | --- | --- | --- | --- | --- | --- | --- | --- |
| Chen, J.(2022) | 1 | 1 | 1 | 1 | 1 | 1 | 1 | 0 | 7 |
| Chu, C. S.(2016) | 1 | 1 | 1 | 1 | 2 | 1 | 0 | 0 | 7 |
| Cui,F.(2019) | 1 | 1 | 1 | 1 | 1 | 0 | 1 | 0 | 6 |
| Feng,D.Y.(2022) | 1 | 1 | 1 | 1 | 1 | 1 | 0 | 0 | 6 |
| Feng,K.(2022) | 1 | 1 | 1 | 1 | 1 | 1 | 0 | 0 | 6 |
| Guan, H. L.(2022) | 1 | 1 | 1 | 1 | 2 | 1 | 1 | 0 | 8 |
| Guan, H. L.(2020) | 1 | 1 | 1 | 1 | 1 | 1 | 1 | 0 | 7 |
| Guo, Y.(2016) | 1 | 1 | 1 | 1 | 2 | 1 | 1 | 0 | 8 |
| Hu,G.L.(2022) | 1 | 1 | 1 | 1 | 2 | 1 | 0 | 0 | 7 |
| Hu,L.(2019) | 1 | 1 | 1 | 1 | 2 | 1 | 0 | 0 | 7 |
| Ji,W.W.(2022) | 1 | 1 | 1 | 1 | 2 | 1 | 1 | 0 | 8 |
| Kong, D.(2022) | 1 | 1 | 1 | 1 | 1 | 1 | 0 | 0 | 6 |
| Kong,S.W.(2021) | 1 | 1 | 1 | 1 | 2 | 0 | 1 | 0 | 7 |
| Lai, C. C.(2022) | 1 | 1 | 1 | 1 | 2 | 1 | 1 | 1 | 9 |
| Li, B. B.(2022) | 1 | 1 | 1 | 1 | 2 | 1 | 1 | 0 | 8 |
| Li,N.(2022) | 1 | 1 | 1 | 1 | 2 | 1 | 1 | 0 | 8 |
| Li, T.(2017) | 1 | 1 | 1 | 1 | 2 | 1 | 0 | 0 | 7 |
| Li, X. W.(2022) | 1 | 1 | 1 | 1 | 2 | 1 | 1 | 0 | 8 |
| Liang, C. K.(2015) | 1 | 1 | 1 | 1 | 1 | 1 | 0 | 0 | 6 |
| Liang,D.S.(2022) | 1 | 1 | 1 | 1 | 1 | 1 | 1 | 0 | 7 |
| Liang,M.(2020) | 1 | 1 | 1 | 1 | 2 | 0 | 1 | 0 | 7 |
| Liao,Y.L.(2019) | 1 | 1 | 1 | 1 | 1 | 1 | 1 | 0 | 7 |
| Lin,B.(2022) | 1 | 1 | 1 | 1 | 1 | 1 | 0 | 0 | 6 |
| Lin, X.(2020) | 1 | 1 | 1 | 1 | 1 | 1 | 1 | 0 | 7 |
| Liu,J.H.(2009) | 1 | 1 | 1 | 1 | 1 | 0 | 1 | 0 | 6 |
| Liu,J.M.(2018) | 1 | 1 | 1 | 1 | 1 | 1 | 1 | 0 | 7 |
| Liu,S.J.(2018) | 1 | 1 | 1 | 1 | 1 | 1 | 1 | 0 | 7 |
| Liu,X.L.(2022) | 1 | 1 | 1 | 1 | 1 | 1 | 1 | 0 | 7 |
| Liu,Y.X.(2017) | 1 | 1 | 1 | 1 | 1 | 0 | 1 | 0 | 6 |
| Shi,M.(2022) | 1 | 1 | 1 | 1 | 2 | 1 | 1 | 0 | 8 |
| Song, J.(2022) | 1 | 1 | 1 | 1 | 1 | 1 | 1 | 0 | 7 |
| Sun,F.P.(2022) | 1 | 1 | 1 | 0 | 1 | 1 | 1 | 0 | 6 |
| Sun,X.S.(2022) | 1 | 1 | 1 | 1 | 1 | 1 | 1 | 0 | 7 |
| Tan,G.(2011) | 1 | 1 | 1 | 1 | 1 | 1 | 1 | 0 | 7 |
| Tsai, C. Y.(2022) | 1 | 1 | 1 | 1 | 1 | 1 | 1 | 0 | 7 |
| Wang,W.(2011) | 1 | 1 | 1 | 1 | 1 | 1 | 1 | 1 | 8 |
| Wu,J.N.(2022) | 1 | 1 | 1 | 1 | 1 | 1 | 1 | 0 | 7 |
| Xiao,Y.Q.(2022) | 1 | 1 | 1 | 1 | 1 | 1 | 1 | 0 | 7 |
| Xing, H.(2020) | 1 | 1 | 1 | 1 | 1 | 1 | 1 | 0 | 7 |
| Xu,H.(2022) | 1 | 1 | 1 | 1 | 1 | 1 | 0 | 0 | 6 |
| Xu,H.C.(2022) | 1 | 1 | 1 | 1 | 1 | 1 | 1 | 0 | 7 |
| Xue, P.(2016) | 1 | 1 | 1 | 1 | 2 | 0 | 1 | 1 | 8 |
| Yu,R.(2016) | 1 | 1 | 1 | 1 | 1 | 1 | 1 | 0 | 7 |
| Yuan,Y.(2018) | 1 | 1 | 1 | 1 | 1 | 1 | 0 | 0 | 6 |
| Yue,H.L.(2022) | 1 | 1 | 1 | 1 | 1 | 1 | 0 | 0 | 6 |
| Zhang, H.(2017) | 1 | 1 | 1 | 1 | 0 | 1 | 1 | 0 | 6 |
| Zhang,H.(2022) | 1 | 1 | 1 | 1 | 1 | 1 | 1 | 0 | 7 |
| Zhang,P.F.(2018) | 1 | 1 | 1 | 1 | 1 | 1 | 1 | 0 | 7 |
| Zhang, Y.(2023) | 1 | 1 | 1 | 1 | 2 | 1 | 1 | 0 | 8 |
| Zhou,L.J.(2020) | 1 | 1 | 1 | 1 | 1 | 1 | 1 | 0 | 7 |
| Zhu,L.(2013) | 1 | 1 | 1 | 1 | 2 | 1 | 1 | 0 | 8 |
| Assessment items：1:Representativeness of the Exposed Cohort(1 point); 2:Selection of the Non-Exposed Cohort(1 point); 3:Ascertainment of Exposure(1 point); 4:Demonstration That Outcome of Interest Was Not Present at Start of Study(1 point); 5:Comparability of Cohorts on the Basis of the Design or Analysis(2 points); 6:Assessment of Outcome(1 point); 7:Was Follow-Up Long Enough for Outcomes to Occur(1 point); 8:Adequacy of Follow Up of Cohorts(1 point). | | | | | | | | | |

Table 2 Quality evaluation of case-control studies

| Author/Year | 1 | 2 | 3 | 4 | 5 | 6 | 7 | Score |
| --- | --- | --- | --- | --- | --- | --- | --- | --- |
| Chen, J.(2022) | 1 | 1 | 1 | 1 | 1 | 1 | 0 | 6 |
| Assessment items：1:Is the Case Definition Adequate?(1 point); 2:Representativeness of the Cases(1 point); 3:Selection of Controls(1 point); 4:Definition of Controls(1 point); 5:Comparability of Cases and Controls on the Basis of the Design or Analysis(2 point); 6:Ascertainment of Exposure(1 point); 7:Non-Response Rate(1 point). | | | | | | | | |
