# Supplementary material for: Incidence of delirium after non-cardiac surgery in the Chinese elderly population: a systematic review and meta-analysis
Source: Front Aging Neurosci. 2023 Jun 29;15:1188967. doi: 10.3389/fnagi.2023.1188967 (PMC10346854; doi:10.3389/fnagi.2023.1188967)
Supplement: Supplementary file 3 [file Table_3.DOCX]

**Appendix C Sensitivity analysis**

Part 1: Graphs of sensitivity analysis of the incidence of delirium after non-cardiac surgery in the elderly in China

Part 2: Results of sensitivity analysis

------------------------------------------------------------------------------

Study omitted | Estimate [95% Conf. Interval]

-------------------+----------------------------------------------------------

Chen, J. (2022) | .18435432 .16243064 .20627801

Chu, C. S. (2016) | .18812063 .16571386 .21052739

Cui,F. (2019) | .18833677 .16595607 .21071747

Feng,D.Y. (2022) | .18905756 .16726615 .21084896

Feng,K. (2022) | .18577932 .16368943 .2078692

Guan, H. L. (2022) | .18439768 .16252275 .20627262

Guan, H. L. (2020) | .18361276 .16181909 .20540644

Guo, Y. (2016) | .18563679 .16347553 .20779806

Hu,G.L. (2022) | .18728606 .16511121 .2094609

Hu,L. (2019) | .18410273 .16219828 .2060072

Ji,W.W. (2022) | .18470286 .16272371 .206682

Kong, D. (2022) | .18728018 .16501874 .20954163

Kong,S.W. (2021) | .18373904 .16200048 .2054776

Lai, C. C. (2022) | .18884969 .1668423 .21085708

Li, B. B. (2022) | .18298057 .16149148 .20446965

Li,N. (2022) | .1832739 .16150169 .20504609

Li, T. (2017) | .18539153 .16329327 .2074898

Li, X. W. (2022) | .18350461 .16168018 .20532905

Liang, C. K. (2015) | .1884048 .16611724 .21069236

Liang,D.S. (2022) | .18609923 .16400012 .20819834

Liang,M. (2020) | .18570982 .16357478 .20784485

Liao,Y.L. (2019) | .18653798 .16429377 .20878221

Lin,B. (2022) | .18433467 .16236384 .20630547

Lin, X. (2020) | .18770866 .16531827 .21009906

Liu,J.H. (2009) | .18857937 .16639456 .21076415

Liu,J.M. (2018) | .1857543 .1636177 .20789091

Liu,S.J. (2018) | .18443069 .16245435 .20640701

Liu,X.L. (2022) | .18715285 .16483566 .20947002

Liu, Y. M. (2022) | .186023 .16370654 .20833945

Liu,Y.X. (2017) | .18704796 .16484597 .20924993

Shi,M. (2022) | .18708722 .16486862 .20930582

Song, J. (2022) | .18662107 .16448121 .20876092

Sun,F.P. (2022) | .18709794 .16441986 .20977601

Sun,X.S. (2022) | .1878487 .16555691 .2101405

Tan,G. (2011) | .18783703 .1652907 .21038337

Tsai, C. Y. (2022) | .18884969 .1668423 .21085708

Wang,W. (2022) | .18352525 .16165408 .20539643

Wu,J.N. (2022) | .18456547 .1629554 .20617555

Xiao,Y.Q. (2022) | .1865371 .16438569 .20868851

Xing, H. (2020) | .18315336 .16137071 .20493603

Xu,H. (2022) | .18657967 .16434655 .20881282

Xu,H.C. (2022) | .18620448 .1639518 .20845714

Xue, P. (2016) | .18841931 .16617696 .21066165

Yu,R. (2016) | .18510272 .16306795 .20713748

Yuan,Y. (2018) | .18802467 .16580132 .21024804

Yue,H.L. (2022) | .18438038 .16240615 .20635462

Zhang, H. (2017) | .18755363 .1652016 .20990565

Zhang,H. (2022) | .18537454 .16328621 .20746288

Zhang,P.F. (2018) | .18752636 .16524598 .20980674

Zhang, Y. (2023) | .18604651 .16392063 .2081724

Zhou,L.J. (2020) | .18498346 .16294223 .20702469

Zhu,L. (2013) | .186299 .16402231 .20857567

-------------------+----------------------------------------------------------

Combined | .18611051 .16422247 .20799855

---------------------------------------------------------------
